# Maternal death and obstetric care audits in Nigeria: a systematic review of barriers and enabling factors in the provision of emergency care

**DOI:** 10.1186/s12978-016-0158-4

**Published:** 2016-04-22

**Authors:** Julia Hussein, Atsumi Hirose, Oluwatoyin Owolabi, Mari Imamura, Lovney Kanguru, Friday Okonofua

**Affiliations:** Immpact, University of Aberdeen, Health Sciences Building, University of Aberdeen, Foresterhill, Aberdeen, AB25 2ZD Scotland; Women’s Health and Action Research centre (WHARC), KM 11 Benin-Lagos Expressway, Igue-Iheya, Benin City, Edo State Nigeria

**Keywords:** Maternal mortality, Maternal death reviews, Audit, Surveillance and response, Quality improvement, Emergency obstetric care

## Abstract

**Background:**

Maternal death reviews and obstetric audits identify causes and circumstances related to occurrence of a maternal death or serious complication and inform improvements in quality of care. Given Nigeria’s high maternal mortality, the lessons learned from past experiences can provide a good evidence base for informed decision making. We aimed to synthesise findings from maternal death reviews and other obstetric audits conducted in Nigeria through a systematic review, seeking to identify common barriers and enabling factors related to the provision of emergency obstetric care.

**Methods:**

We searched for maternal death reviews and obstetric care audits reported in the published literature from 2000–2014. A ‘best-fit’ framework approach was used to extract data using a structured data extraction form. The articles that met the inclusion criteria were assessed using a nine point quality score.

**Results:**

Of the 1,841 abstracts and titles at initial screening, 329 full text articles were reviewed and 43 papers fulfilled the inclusion criteria. Four types of barriers were reported related to: transport and referral; health workers; availability of services; and organisational factors. Three elements stand out in Nigeria as contributing to maternal mortality: delays in Caesarean section, unavailability of magnesium sulphate and lack of safe blood transfusion services.

**Conclusions:**

Obstetric care reviews and audits are useful activities to undertake and should be promoted by improving the processes used to conduct them, as well as extending their implementation to rural and basic level health facilities and to the community. Urgent areas for quality improvement in obstetric care, even in tertiary and teaching hospitals should focus on organisational factors to reduce delays in conducting Caesarean section and making blood and magnesium sulphate available for all who need these interventions.

## Background

Maternal death reviews and obstetric audits are quality improvement investigations which support the identification and analysis of causes and circumstances related to occurrence of maternal deaths or serious complications. They have been conducted for many years in various settings, although there have been recent efforts to promote their implementation, especially in low and middle income countries [1, 2]. Recently, the World Health Organization produced a guideline for conducting maternal death surveillance and response (MDSR), which builds on maternal death reviews and emphasises the continuous action cycle and ongoing monitoring necessary to link the health information system with quality improvement processes [3]. From a public services perspective, audit can be seen as a social process, which allows checking and verification, providing evidence to reduce problem areas [4, 5].

As a consequence of the size of its population, Nigeria is the African country with the highest contribution to maternal deaths, making up 14 % of the 289,000 annual global maternal deaths [6]. Although maternal mortality has fallen from 800 deaths per 100,000 live births in 2003 to 545 deaths per 100,000 live births in 2008 [7], the most recent estimates show that progress is slow. The maternal mortality in 2013 was 576, compared to a previous estimate in 2008 of 545 deaths per 100,000 live births [8]. At policy level, there is clear commitment in Nigeria to improve maternal health and reduce maternal mortality, with various national initiatives being implemented at national and state levels such as health insurance programmes, community health worker development and improvements in midwifery services [6, 9–12].

Rational and effective use of reliable, routinely collected data will result in better information for planning and action as well as generate an accountable and responsive health service. Conducting maternal death reviews and other types of obstetric audit are a way to achieve this pathway to change. The audits and reviews assess clinical practices to report health outcomes, highlight deficiencies in service provision, provide recommendations for improvements in care and are an established means to improve the quality of maternity care [1, 13–15] In recognition of these attributes, in 2013 the Ministry of Health in Nigeria, Society of Gynaecology and Obstetrics of Nigeria (SOGON) and International Federation of Gynaecology and Obstetrics (FIGO) embarked on a process to commence a national maternal death review programme [16]. Yet Nigeria already has a long history of conducting maternal death reviews and obstetric audit. Most have been conducted independently in individual hospitals, rather than as part of a structured programme. There have been no attempts to collectively summarise past experiences [17] which may strengthen the evidence base for decision-making beyond that of individualised efforts. In contribution to the efforts of SOGON and the Ministry of Health in Nigeria, we set out to synthesise the findings of previous maternal death reviews and other obstetric audits through a systematic review. Specifically, we sought to capture commonly encountered barriers and to identify enabling factors related to the provision of emergency obstetric care.

## Methods

### Eligibility criteria

A systematic search of the published literature was conducted using a pre-defined protocol. We used a broad definition of ‘audit’ and ‘reviews’ [13] to include studies and surveys which used clinical case records, organisation of clinical meetings or interviews to report on obstetric health care in Nigeria. For the sake or brevity, we will use the term ‘audit’ in the remainder of this paper. Studies using either numerical or qualitative data, or both, to record parameters associated with care provision were included. The population of interest were women who experienced any emergency obstetric complication which was life-threatening for the women themselves (including death), required emergency surgery as a consequence of the complications, or was life threatening for their babies. Studies could be undertaken at community, healthcare facility, or at district, regional or national level. The primary outcomes of interest were circumstances surrounding maternal deaths and complications after women sought care, including referral and transport; descriptions of clinical practice, treatment or management of women experiencing maternal mortality or complications; and descriptions of health professional performance or practice in a healthcare setting in relation to women during pregnancy and childbirth.

Studies with the following characteristics were excluded: documentation of causes or proportions of maternal deaths without descriptions of care practices or management; reports focusing exclusively on routine care (e.g., antenatal care, partogram use) with no obstetric complications; sole focus on socio-demographic risk factors (e.g., age, parity) and those that evaluated only knowledge, attitudes or satisfaction of pregnant women, their families or healthcare providers. Management of non-emergency conditions such as chronic obstetric fistula, congenital malformations or those requiring elective surgery and other procedures are excluded. Studies not located in Nigeria, even if they involved women from Nigeria, were excluded.

### Search methods

Medline; Embase; CINAHL; Web of Knowledge and African Journal Online were searched in December 2013 and January 2014 using terms related to maternal and perinatal mortality and complications; setting (Nigeria) and study design (audit/confidential enquiry/review/survey). We restricted our search to studies published during or after the year 2000 because we were interested in contemporary practices and because Nigeria has seen considerable effort put into national strategies and interventions since 2000 [11]. Studies published after the year 2000, but which included data prior to the year 2000 were not included unless there was a means of separating the post-2000 data from previous years. There were no language restrictions imposed on the searches. Bibliographic references from the included studies were reviewed to identify additional studies.

### Data collection and analysis

All titles, abstracts and full-text articles were reviewed by at least one reviewer and a sample checked by another reviewer. Disagreements were resolved by discussion. Data was extracted from included studies by one reviewer and checked by a second reviewer.

Data was synthesised based on a ‘best-fit’ framework approach [18, 19] as follows. A structured data extraction form, which reflected a pre-set thematic framework was used to extract data. The framework originated from the South African Confidential Enquiry format. Its structure was made up of categories related to patient factors, the clinical management of emergency complications health system factors such as transport, referral, health personnel and their training, the availability of services and organisational/administrative factors such as record keeping and policy or planning factors [20, 21]. Patient factors were analysed separately and reported elsewhere.

The focus of the review was to identify barriers and enabling factors reported in maternal death audits of obstetric emergencies.

### Quality assessment

The articles that met the inclusion criteria were assessed using a nine point score adapted from others [1, 22]. A score of 0 or 1 was given for each study characteristic as follows: description of study population, explanation of sampling strategy, consideration of missing cases, pretesting or piloting of study instruments, use of a standard data collection form, description of data collectors, training, quality checks for data entry and assessment of inter-observer/inter-site variability. Two reviewers conducted the quality assessment independently, their assessments were compared and disagreements resolved by discussion.

## Results

**Fig 1:**
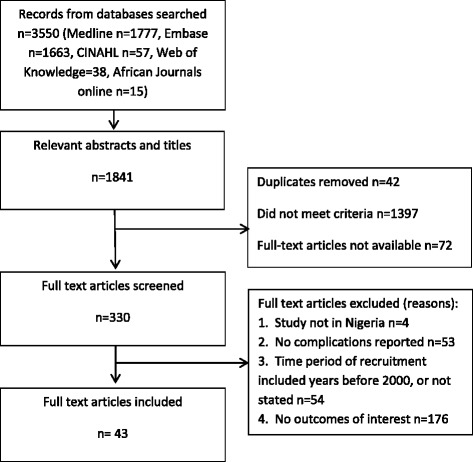
Search Results. Flow chart of search, screening, included papers and reasons for exclusion

From the 1,841 relevant abstracts and titles found, 329 full text articles were retrieved (Fig. 1). All were in English. Of these, 43 papers fulfilled the inclusion criteria [23–65]. Two papers used the same data but analysed the findings from different viewpoints, so we merged these two papers as one [27, 28].

The included papers and their characteristics are summarised in Table 1, categorised according to the complication or obstetric outcome of interest. The largest number of studies included were of eclampsia/pre-eclampsia and Caesarean sections. All studies were hospital based and the six geopolitical zones of Nigeria were represented in the included papers. The purpose of most of the studies were to ascertain or compare obstetric outcomes or practices and to provide recommendations for improved care. Studies made comparisons between cases and controls [30], hospitals [53], procedures [58], across time periods [27] or against rates of complications reported in other studies [35, 38, 47, 61]. A few conducted audits against pre-specified criteria or standard treatment protocols [27–30]. All but two studies [30, 53] were conducted in a single hospital. Two-thirds of the studies collected data retrospectively over a period of between one to ten years. Prospective studies were fewer in number and covered shorter periods of time from one to three years. Apart from using clinical case notes and other types of hospital case records (such as labour ward registers or admission books) as data sources, some studies used interviews and observation as an alternative [26, 32, 53]. A few papers did not fully describe the means of data collection [49, 54, 60], although the type of data reported suggested that case records were used. Quality scores ranged from 2–7 from a maximum of 9 points (Table 1).

**Table 1:** Study characteristics

Author, year	State/zone	Recruitment years	Setting	Study methods	Numbers of cases included	Numbers of maternal deaths reported	Quality score
Maternal deaths							
Ande 2012 [23]	Benin, SS	2005–2009	1 teaching hospital	Retrospective case record review	184	184	2
Ezugwu 2009 [24]	Enugu, SE	2004–2008	1 teaching hospital	Retrospective case record review	54	54	2
Ozumba 2008 [25]	Enugu, SE	2003–2005	1 teaching hospital	Case record review	47	47	4
Omo-Aghoja 2010 [26]	Benin, SS	2005–2007	1 teaching hospital	Retrospective case record review, inspection of maternity unit and interview of healthcare providers	84	84	4
Near miss							
Oladapo 2005 [27], Oladapo 2007 [28](same data for both papers)	Sagamu, SW	2002–2004	1 teaching hospital	Retrospective case record review, clinical review panel	255	44	3
Hunyinbo 2008 [29]	Abeokuta, SW	2002–2003	1 teaching hospital	Case record review	130	4	4
Adeoye 2013 [30]	Ile Ife, SW	2006–2007	2 teaching hospitals	Prospective case record review	75	nr	7
Olagbuji 2012 [31]	Benin, SS	2007–2010	1 teaching hospital	Retrospective case record review	263	34	2
Stillbirths							
Ezugwu 2011 [32]	Enugu, SE	2009	1 teaching hospital	Prospective case record review and interview of mothers	153	nr	3
Olusanya 2009 [33]	Lagos, SW	2005–2007	1 teaching hospital	Case record review	602	nr	2
Abortion complications							
Ibrahim 2012 [34]	Niger Delta, SS	2007–2010	1 teaching hospital	Retrospective case record review	63	3	2
Raibu 2009 [35]	Lagos, SW	2005–2007	1 tertiary hospital	Prospective case record review	175	nr	3
Nwogu-Ikijo 2007 [36]	Enugu, SE	2000–2005	1 teaching hospital	Retrospective case record review	11	11	2
Ekanem 2009 [37]	Calabar, SS	2003–2004	1 teaching hospital	Prospective case record review	126	5	3
Eclampsia/pre-eclampsia							
Adinma 2013 [38]	Nnewi, SE	2000–2009	1 teaching hospital	Retrospective case record review	46	8	2
Eke 2011 [39]	Anambra, SE	2004–2009	1 tertiary hospital	Case record review	212	nr	2
Agida 2010 [40]	Abuja, NC	2005–2008	1 teaching hospital	Retrospective case record review	46	5	2
Kulima 2009 [41]	Nguru, NE	2003–2007	1 tertiary hospital	Retrospective case record review	224	52	2
Okafor 2008 [42]	Abuja, NC	2001–2005	1 tertiary hospital	Retrospective case record review	38	11	3
Makinde 2009 [43]	Ile Ife, SW	2006	1 teaching hospital	Prospective case record review	34	4	2
Uterine rupture							
Nyengidiki 2011 [44]	Niger delta, SS	2004–2007	1 teaching hospital	Retrospective case record review	40	7	2
Esiki 2011 [45]	Ebonyi, SE	2000–2009	1 teaching hospital	Retrospective case record review	101	12	3
Osaikhuwuomwan 2011 [46]	Niger delta, SS	2003–2007	1 teaching hospital	Retrospective case record review	33	2	2
Okafor 2006 [47]	Enugu, SE	2000–2004	1 teaching hospital	Retrospective case record review	23	1	2
Mbamara 2012 [48]	Nnewi, SE	2004–2009	1 teaching hospital	Retrospective case record review	25	3	3
Caesarean section							
Onankpa 2009 [49]	Sokoto, NW	2006–2007	1 teaching hospital	Prospective, unclear data source	216	nr	3
Ekanem 2008 [50]	Calabar, SS	2000–2001	1 teaching hospital	Retrospective case record review	349	12	2
Ozumba 2006 [51]	Enugu, SE	2000–2002	1 teaching hospital	Retrospective case record review	463	nr	2
Ojiyi 2012 [52]	Orlu, SE	2004–2008	1 teaching hospital	Retrospective case record review	358	3	2
Onah 2005 [53]	Enugu, SE; Abuja, NC	2003	2 hospitals	Prospective observation of events	224	nr	5
Faponie 2007 [54]	Ile Ife, SW	2005	1 teaching hospital	Prospective, unclear data source	641	2	2
Peripartum hysterectomy							
Abasiattai 2013 [55]	Uyo, SE	2004–2011	1 teaching hospital	Retrospective case record review	28	4	2
Nwobodo 2012 [56]	Sokoto, NW	2005–2010	1 teaching hospital	Retrospective case record review	74	9	2
Rabiu 2010 [57]	Lagos, SW	2003–2007	1 teaching hospital	Retrospective case record review	57	11	2
Badjoko 2013 [58]	Ile Ife, SW	2001–2010	1 teaching hospital	Retrospective case record review	55	10	3
Omole Ohinsi 2012 [59]	Kano, NW	2003–2008	1 teaching hospital	Retrospective case record review	30	4	4
Other post partum complications							
Mutihir 2011 [60]	Jos, NC	2005–2008	1 teaching hospital	Prospective, unclear data source	246	nr	3
Ajenifuja 2010 [61]	Ile Ife, SW	2002–2006	1 teaching hospital	Retrospective case record review	112	6	2
Agwu 2008 [62]	Ebonyi, SE	2003–2006	1 teaching hospital	Retrospective case record review	30	nr	2
Miscellaneous complications							
Adelaja 2011 [63]	Sagamu, SW	2005–2007	1 teaching hospital	Retrospective case record review (obstetric emergencies)	262	17	2
Kalu 2011 [64]	Ebonyi, SE	2001–2007	1 teaching hospital	Retrospective case record review (umbilical cord prolapse)	46	nr	2
Lawani 2013 [65]	Ebonyi, SE	2002–2012	1 teaching hospital	Retrospective case record review (ectopic pregnancy)	205	3	4

### Barriers encountered

Four types of barriers were reported, summarised in Table 2. They related to: transport and referral; health workers; availability of services; and organisational factors.

**Table 2:** Summary of reported barriers and enabling factors

Barriers	Article reference	Enabling factors	Article reference
Transport and referral			
Mismanagement by referring provider	[23, 25, 26, 32–38, 44–46, 48, 57, 61]		
Inefficient ambulance services	[26, 62]		
Lack of transport at night	[30, 55]		
Health workers			
Lack of manpower	[26, 28, 32, 49, 53]	Frequent training for clinical procedures	[27, 47]
Unskilled health professionals	[23, 26, 27, 29, 31, 35, 37, 47, 49, 50, 52, 53, 56, 58, 60]		
Low staff morale	[25, 29]		
Availability of supporting services and facilities			
Lack of operation theatres and intensive care units	[26, 53]	Round the clock services	[26, 53]
Lack of specialist medical equipment and services	[25, 26, 42, 65]	Blood banking	[26, 35, 47, 51, 53, 59]
Unavailability of essential drugs and supplies including blood and magnesium sulphate	[25–29, 39–43, 53, 54, 63]		
Unreliable telephone communications and power supply	[26, 28, 53]		
Organisation of care			
Poor record keeping	[24, 38, 45, 64, 65]	Conducting maternal death reviews	[27–29]
Teamwork	[26, 53]	Emergency treatment before requiring payment	[47]
Lack of planning for organisational change	[32, 53]		

#### Transport and referral

Ineffective and inappropriate referral were identified in the studies as important contributory factors to adverse outcomes. Referred obstetric emergencies were reportedly poorly managed prior to arrival. Practitioners responsible for mismanagement of referral and causing delays included traditional birth attendants [23, 44], general practitioners, private providers and unregulated medical providers [25, 33]. These practitioners were based in traditional maternity homes, churches and faith clinics [35, 46, 61] as well as within the routine health system in health facilities [38]. Reported mismanagement prior to arrival in the study hospital included unhygienic birth practices [35], manipulative procedures performed by unqualified people [34–36] and self-induced manipulation [37]. The injudicious use of oxytocin in labor was also described [32, 44, 45, 48]. Some studies described inefficient ambulance services, poor transport between health facilities [26, 62] and lack of transport especially at night [30, 55].

#### Health workers

The availability, skills and morale of health personnel were reported as key barriers to providing adequate care. Lack of manpower was identified [32], including insufficient numbers of nurses and midwives for the existing care loads [26] and absence of middle grade medical officers (registrars) [49]. Junior doctors were reportedly inexperienced and untrained [49, 53] and a number of studies reported delays in provision of appropriate care after the pregnant woman had arrived in the hospital [23, 26, 53]. One study reported that 32.9 % of critically ill women received treatment over an hour after diagnosis had been made [28]. Nursing staff did not perform well in routine monitoring of vital signs [29]. Poor diagnostic skills were a problem, for example with junior doctors and midwives on night duty missing cases of retained placenta [60], poor asepsis practised during procedures [35] and incorrect diagnosis before major surgery [58]. Deviations from standard treatment norms were reported in over 40 % of adverse maternal outcome [28]. Some studies reported a higher occurrence of morbidity during surgery by less experienced obstetricians and anaesthesiologists [37, 49, 56] although others acknowledged that the senior doctors performed more elective procedures which had lower rates of morbidity compared to emergency procedures done by junior and mid-level clinicians [52]. Poor staff morale and motivation were cited, with delays in receiving salaries and unwillingness to follow clinical protocols [29]. Industrial strike action by health workers was reported as contributing to maternal death [25].

#### Availability of supporting services and facilities

Insufficient availability of operation theatres and suboptimal intensive care units were described [26]. Mean decision to delivery intervals for Caesarean sections were 3.3 h in one hospital and 8.5 h in another [53]. Anaesthesia-related factors and poor blood availability were reported in several studies. For instance, delays in proceeding with surgery occurred due to lack of skilled anaesthetists, anaesthetic drugs, gases and equipment; as well as poor availability of blood and cross matching facilities and linen [25, 26, 28, 29, 53, 54, 63]. Other services and facilities reported as lacking included intensive clinical monitoring procedures like arterial blood and central venous pressure [42] and equipment like laparoscopy and ultrasound scanning for diagnosis and treatment [65]. The unavailability of magnesium sulphate was reported in several studies [27, 29, 39–43]. Apart from the clinical services, erratic and unreliable telephone services [53] and power supplies [28] were reported.

#### Organisation of care

Limitations were found in relation to record keeping, procedural norms and planning for change. Examples of poor record keeping included no recording of time from diagnosis to treatment [64] and missing records [24, 38, 45, 65]. The lack of interdepartmental teamwork [26] and poor organisation of rotas (despite availability of personnel) [53] were attributed to causing substandard care. In one review of maternal deaths, a sudden increase in maternal mortality had been observed after the hospital was upgraded from general to specialist level. The authors explained the phenomena as a result of manpower shortages and inadequate facilities to cope with the upgrade, cautioning that careful planning was required to mitigate against problems [32].

### Enabling factors

Specialised and frequent training in abortion care was linked to good management of abortion related complications [27]. Highly experienced anaesthetists reportedly contributed to lowered maternal mortality in emergencies [47]. The availability of 24 h, round the clock medical and laboratory services, with organised staff rotas were cited as contributing factors when care was judged to be adequate in the included studies [26, 53]. The availability of blood bank facilities were cited as an enabling factors in several studies [26, 35, 47, 51, 53]. Conducting audit was found to be a positive experience that led to practical action and improvement in care practices [27–29]. The practice of treating emergencies before demanding payment was also highlighted [47].

## Discussion

### Improving care and reducing maternal mortality

Barriers related to referral, transport, the availability and skills of health professionals, physical infrastructure, lack of essential drugs and supplies, poor record keeping systems and disorganised planning were common factors found across the studies. There were few enabling factors reported. Intensive training for clinical procedures, 24 h services, conducting maternal death reviews and flexible payment modalities reportedly contributed to facilitating care.

The barriers identified concur with the findings of other studies in Africa as well as in other low income settings [66–68]. In a review of audit-identifiable avoidable factors in low resource settings, substandard practices by health workers and delays in blood transfusion were found to be the commonest reported problems [1]. Of the priority emergency obstetric services defined by the World Health Organization [69], three elements stand out in Nigeria: delays in Caesarean section, unavailability of magnesium sulphate and lack of safe blood transfusion services. We found that these three situations occur in tertiary and teaching hospitals in Nigeria, yet the facilities reporting their occurrence represent the pinnacle of service provision and it is likely the deficiencies are even more pronounced in rural and basic-level hospitals.

With a quarter of a century of safe, low cost magnesium sulphate use behind us, it is inexcusable that any country still experiences unavailability. Problems of production and distribution, inadequate and poorly implemented clinical guidelines, and the lack of political support for policy change need to be addressed [70]. In 2004, Nigeria was one of the recipients of the the US President’s Emergency Plan for AIDS Relief (PEPFAR) programme which aimed to strengthen national blood transfusion services in the setting of high HIV prevalence, yet calls to take action continue [71] . Our study gives weight to an urgent need for national and local efforts to resolve these fundamental shortcomings in the availability of blood and magnesium sulphate. Delays experienced in conducting emergency caesarean sections are analysed in depth by several authors of the included papers [28, 37, 53]. The challenges faced are complex and relate to a range of health system factors including availability, skills and skill mix of the obstetric team and organisation of services. Similarly, addressing the problems of transport, referral and record keeping require a health systems approach, further probing into root causes and combinations of solutions and actions both at local and central level.

### Good practices for conducting obstetric audit

**Fig 2:**
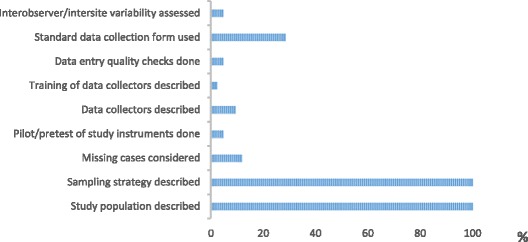
Percentage of included papers fulfilling quality assessment criteria. Graph showing percentage of papers included in the systematic review which fulfilled each of nine quality assessment criteria

Apart from our aim of identifying barriers and enabling factors, this systematic review has drawn out important considerations related to improving how maternal death reviews and obstetric audits are conducted. In the case of Nigeria, this will have particular relevance given the mandate to roll-out a national programme. Given the years of experience found in Nigeria, the aim should be not only to promote the conduct of audit in settings outside the tertiary and teaching hospitals, but also to improve the way audits are conducted so as to implement a process that captures insights and problems more effectively. In this systematic review we found a few studies which explicitly made comparisons against pre-specified criteria [27–30], which allows for a structured and objective assessment of care against explicit criteria, making for improved utility as a quality improvement tool [1, 13]. Other studies found also provided useful insights into aspects of quality improvement, although the process through which their findings were reported could be haphazard and sometimes anecdotal. In addition, the quality assessment we used highlighted a number of good practices we think useful to consider in improving the conduct and reporting of audits and reviews. Of the nine criteria we used in our quality assessment, all papers described the study population and sampling process but other good practices such as training for data collectors and standardised data entry (Fig. 2) were not described by authors, and we surmise that they were not done. The presentation of maternal death reviews could be improved by paying greater attention to describing these criteria fully. Underlying these design and reporting issues are the need to improve case notes and reduce the proportion of missing information in the routine health information system.

### Limitations

We faced some limitations in conducting the systematic review. Our search was confined to databases of published literature. Maternal death reviews done may not be published or may be reported in journals with incomplete archives, as evidenced by the relatively large number (*n* = 72) of full-text articles not available on-line or on inter-library loan from the British Library (Fig. 1). To capture a wider range of studies, we used broader inclusion criteria to define maternal death reviews and audits. Nevertheless our sample may not have been exhaustive. However, the purpose of synthesis in this review was to seek coherence of findings and to provide interpretive explanation. As similar barriers were found across the different and independent studies across Nigeria we suggest that commonly experienced factors are likely to have been captured [72]. Our study was confined in terms of its representativeness of different types of health facilities. All studies found were conducted in tertiary or teaching hospitals, so the findings cannot be extrapolated to the circumstances and care provided in primary and secondary level health facilities in Nigeria. With 36 % of deliveries occurring in health facilities in Nigeria and 38 % with health professionals [8] the services provided at tertiary level represent only the ‘tip of the iceberg’. It may be argued that critically ill women will present at tertiary facilities however, the reality is that many women in low and middle income countries are unable to reach this level of the health system [68]. It is likely that the barriers faced are greater in other types of health facilities, as the teaching and tertiary hospitals represent the best care available to the majority of women. In addition, the hospital based studies do not permit full elicitation of community factors that impact on emergency care. Despite these caveats, we believe useful lessons can be drawn from this systematic review.

## Conclusion

This systematic review synthesises experiences of maternal death review and other obstetric audits in Nigeria. The barriers identified are avoidable and most can be overcome by strong and concerted action.

The recommendations emerging from this review are of three types: improving care, enhancing the existing audit process, and extending audit beyond the tertiary and teaching hospital setting. For the improvement of emergency obstetric care, it is clear that concerted effort is needed to make magnesium sulphate for eclampsia consistently available throughout the country and to improve blood transfusion services. Organisational change should especially address delays in conducting emergency Caesarean sections, referral barriers and manpower problems in the health system. The new national maternal death review system in Nigeria can take cognisance of the experience already built up and place emphasis on improving how audits are conducted. To enhance existing audit processes, we suggest that, by setting pre-specified criteria and making comparisons against these criteria, assessments of care may be improved by becoming more structured and objective I n nature. Training for those involved in audit especially individuals responsible for reporting and collecting data will be necessary, along with the need to improve on how events are recorded in clinical case notes. The storage and retrieval of case notes is also important to reduce the proportion of missing information. Finally, our knowledge of care practices is confined to the highest referral level. We recommend the extension of reviews and audits to other health facilities and in the community. This could be done initially by setting up well conducted small scale studies to learn from implementation of audit outside hospital environments: the problems are likely to be similar but more pronounced to those seen in hospitals, such as poor record keeping, lack of experience in conducting audits and low staff motivation. Identifying a suitable site to conduct pilot audit studies will also contribute to the learning experience before attempting to scale up efforts. Experiences will be gained by targeting various settings such as district hospitals, health centres and community-based locations, with each having a set of different barriers to overcome.

This synthesis has shown that the key challenges to reducing maternal mortality in Nigeria are already well known and that we also have the know-how to implement the required actions. ‘Business as usual’ is not enough. The challenge for the future is to build on the experiences of the past, deepen our understanding, generate creative solutions for complex problems and persist in implementing what we know should be done.
